# Tumor response and outcome after reverse treatment for patients with synchronous colorectal liver metastasis: a cohort study

**DOI:** 10.1186/s12893-020-00738-3

**Published:** 2020-04-19

**Authors:** Céline Du Pasquier, Didier Roulin, Pierre Bize, Christine Sempoux, Caterina Rebecchini, Michael Montemurro, Markus Schäfer, Nermin Halkic, Nicolas Demartines

**Affiliations:** 1 Department of Visceral Surgery, Lausanne University Hospital (CHUV), University of Lausanne, Rue du Bugnon 46, 1011 Lausanne, Switzerland; 2 Department of Radiology, Lausanne University Hospital (CHUV), University of Lausanne, Rue du Bugnon 46, 1011 Lausanne, Switzerland; 3 Department of Pathology, Lausanne University Hospital (CHUV), University of Lausanne, Rue du Bugnon 46, 1011 Lausanne, Switzerland; 4 Department of Medical Oncology, Lausanne University Hospital (CHUV), University of Lausanne, Rue du Bugnon 46, 1011 Lausanne, Switzerland

**Keywords:** Colorectal liver metastases, Reverse treatment, Liver-first, Liver surgery, Neoadjuvant chemotherapy

## Abstract

**Background:**

The reverse treatment of patients with synchronous colorectal liver metastases (CRLM) is a sequential approach with systemic chemotherapy first, followed by liver resection, and finally, primary tumor resection. The aim of this study was to assess the feasibility, the radiological and pathological tumor response to neoadjuvant therapy, recurrence rates and long-term survival after reverse treatment in a cohort study.

**Methods:**

Data from patients with CRLM who underwent a reverse treatment from August 2008 to October 2016 were extracted from our prospective hepato-biliary database and retrospectively analyzed for response rates and survival outcomes. Radiological tumor response was assessed by RECIST (Response Evaluation Criteria In Solid Tumor) criteria and pathological response according to TRG (Tumor Regression Grade). Disease-free and overall survival were estimated with Kaplan-Meier survival curves.

**Results:**

There were 44 patients with 19 rectal and 25 colonic tumors. The reverse treatment was fully completed until primary tumor resection in 41 patients (93%). Radiological assessment after chemotherapy showed 61% of complete/partial response. Pathological tumor response was major or partial in 52% of patients (TRG 1–3). Median disease-free survival after primary tumor resection was 10 months (95% CI 5–15 months). Disease-free survival at 3 and 5 years was 25% and 25%, respectively. Median overall survival was 50 months (95% CI 42–58 months). Overall survival at 3 and 5 years was 59% and 39%, respectively.

**Conclusion:**

The reverse treatment approach was feasible with a high rate of patients with complete treatment sequence and offers promising long-term survival for selected patients with advanced simultaneous colorectal liver metastases.

## Background

At the time of diagnosis, up to 20% of patients with colorectal cancer have simultaneous liver metastases [1]. In addition to treat the primary tumor, complete resection of colorectal liver metastases (CRLM) is mandatory to provide a curative treatment [2]. Development of new anti-cancer drugs and their combination with anti-VEGF and/or anti-EGFR agents increased the major tumor response rate up to 72% [3–7], thus offering to new treatment strategies. High response rates to chemotherapy offer the potential of curative treatment after downsizing CRLM for patients with initial unresectable disease. The traditional treatment (“classic”) consists in a staged approach with resection of the primary colorectal tumor, followed by systemic chemotherapy and liver resection.

The reverse treatment or liver-first approach, first described by Mentha in 2006, was based on initial neoadjuvant chemotherapy, followed by liver resection and finally, primary tumor resection [8]. The rationale of this strategy was to target liver metastases first, thus avoiding their progression during treatment of the primary tumor, especially for patients with multiple or large CRLM. The precise selection criteria for choosing classical or reverse strategies were not clearly defined, and none of these have until now shown any survival advantage [9]. The reverse treatment approach has been used at our institution since 2008. The study aim was to assess the feasibility and completion rate of liver-first treatment, radiological and pathological tumor response to neoadjuvant chemotherapy, as well as overall and disease-free survival.

## Methods

### Study design

Retrospective analysis of prospectively collected data on consecutive patients who underwent liver resection for CRLM within a reverse treatment at our institution from August 2008 to October 2016. Selection criteria for reverse treatment was synchronous CRLM with predominant hepatic disease with no bowel occlusion at initial presentation as evaluated and validated during multidisciplinary tumor board. Exclusion criteria for a liver-first approach were: metastases not located in the liver or lung, persistent unresectable liver metastases after neoadjuvant chemotherapy, and more than 3 pulmonary metastases. Patients with refusal of consent were not included and written consent was obtained for all participants.

This study was approved by the local ethical committee (2016–01286) and conducted in accordance with the STROBE criteria (http://strobe-statement.org/).

### Treatment strategy

A reverse treatment was defined as sequential management with neoadjuvant chemotherapy, hepatic resection, and then resection of the primary colorectal cancer. Following neoadjuvant chemotherapy, radiological reassessment with contrast-enhanced CT and/or liver MRI was performed after 2 to 6 cycles of treatment to evaluate the response of CRLM according to the response evaluation criteria in solid tumors (RECIST) [10]. Liver resections were performed by experienced hepatobiliary surgeons with curative intent. Intraoperative ultrasound was used to guide the resection. Primary tumors were resected after complete hepatic clearance. In case of pulmonary metastases, they were treated after hepatic and primary tumor resection. The need for adjuvant chemotherapy was discussed at our multidisciplinary tumor board. The oncological follow-up was carried out by oncologists or surgeons, with at least monitoring of CEA, thoraco-abdominal CT every 3 months for the first year, and then every 6 months and liver MRI every 6 months.

### Outcome measures

Postoperative complications within 30 postoperative days were prospectively collected in our hepatobiliary and colorectal databases. Mortality was assessed after 30 and 90 postoperative days. The Clavien classification [11], grading the most severe complication of each patient, and the Comprehensive Complication Index (CCI) [12], an index calculated by adding each complication weighted for its severity, were reported. Major complications were defined as Clavien grade 3 or 4.

Radiological response to neoadjuvant treatment according to RECIST criteria was systematically reevaluated by a senior radiologist (PB). Pathologic data were independently re-analyzed for purpose of the present study by two pathologists, one senior with gastro-intestinal and hepatobiliary expertise (CS) and one junior involved in gastro-intestinal and hepatobiliary pathology (CR). All archival slides (from formalin-fixed paraffin-embedded tissue) were reviewed, blinded from the rest of the study. Forty-four cases were analyzed, from which four consultation cases and three local cases had no colorectal resection material available.

For the liver metastasis specimens, tumor regression grade (TRG) was assessed in each metastasis according to Rubbia-Brandt [13]. Non-tumoral liver parenchyma was analyzed to define the presence of chemotherapy associated liver injury. The presence of sinusoidal obstruction syndrome (SOS) was graded according to Rubbia-Brandt [14]. The presence of steatosis and steato-hepatitis was evaluated and fibrosis was graded according to the METAVIR score [15].

For the colorectal resections specimens, TRG was assessed according to Mandard (TRG 1 to 5, with TRG 1 corresponding to complete regression with absence of histologically identifiable residual cancer and fibrosis) [16]. In the peri-tumoral region, the amount of fibrosis and inflammation was evaluated in 3 grades (1: low; 2: moderate 3: abundant). For patients with multiple liver metastases and different TRG, the worse metastasis (lowest response) was used for TRG categorization [13].

### Follow-up method

Follow-up was made on a medical chart basis. In case of missing data due to outwards follow-up of the patient, the treating oncologist was contacted by mail for an update. Disease-free survival (DFS) was calculated from the day of the primary cancer resection. Overall survival (OS) was calculated from the day of the primary diagnosis.

### Statistical analysis

Categorical variables were reported as numbers and percentages, while continuous variables were reported as medians and interquartile ranges for non-normally distributed data, or means and standard deviations for normally distributed data. DFS and OS were calculated with the Kaplan-Meier method. Statistical analysis was made with SPSS statistical software package (SPSS version 23 for windows, SPSS inc., Chicago, IL, USA).

## Results

### Patients’ characteristics and treatment modalities

A total of 44 patients underwent liver resection in the setting of reverse treatment; demographics and characteristics are shown in Table 1. Most patients (33/44, 75%) initially had unresectable CRLM, as assessed by HPB surgeons based on location, size of metastasis and Future Remnant Liver calculation (FRL).

**Table 1:** Preoperative characteristics of patients with synchronous CRLM and selected for a reverse strategy

		*N* = 44	
Age (y, median)		63	Range 23–78
Gender (M: F)		28: 16	
Primary tumor location	Colon	25	57%
	Rectum	19	43%
Number of CRLM (median)		5	Range 1–30
Bilobar liver disease		30	68%
Size of largest CRLM (mm, median)		50	Range 9–151
Initial CEA (μg/l, median)		24.8	Range 0.6–1300

*CRLM* colorectal liver metastasis; *CEA* carcinoembryonic antigen.

Chemotherapy regimen were decided by the referring oncologist on an individualized basis. Patients received a median of six cycles (range 2–12) of neoadjuvant chemotherapy, Oxaliplatin or Irinotecan-based (13 FOLFOX, 15 FOLFIRI, 4 FOLFIRINOX, 4 XELOX, 8 OCFL), with adjunction of anti-VEGF antibody (bevacizumab) in 19 patients (43%) and anti-EGFR antibody (cetuximab) in 16 patients (36%). Three patients (7%) received both bevacizumab and cetuximab. Thirteen (30%) patients were initially treated with palliative chemotherapy and referred to our center because of good response to treatment. Radiological reassessment was performed after a median of 4 cycles of chemotherapy (range 2–6) with chest and abdominal CT scan and liver MRI.

Eighteen patients (41%) needed portal vein embolization to increase FRL volume. One patient underwent simultaneous hepatic vein and portal vein embolization. For additional small metastases, 11 patients underwent thermoablation (radiofrequency or microwave), either preoperatively (*n* = 3, 6%), during liver surgery (*n* = 5, 11%) or both (*n* = 3, 6%).

Three patients (7%) presented pulmonary metastases at the time of diagnosis. Neoadjuvant chemotherapy induced complete response of the lung lesions in 2 patients. The third patient underwent lung wedge resections following resection of the primary tumor.

### Liver-first treatment feasibility

Forty-one out of 44 patients (93%) completed the treatment until primary tumor resection. The three patients who could not complete the treatment had early metastatic recurrence. The median interval between the end of chemotherapy and liver resection was 5.7 weeks (range: 2.1–19.7 weeks). Longer intervals were seen for patients referred by external oncologists after good response to palliative chemotherapy. For patients who underwent the whole treatment, median interval between liver and colorectal surgery was 9.7 weeks (range: 3.5–34.1 weeks).

Bowel occlusion was experienced by five patients (11%). Two patients needed tumor stent placement and another two needed diverting stoma because of occlusive symptoms during neoadjuvant chemotherapy. One patient needed a Hartmann’s procedure for bowel occlusion after liver resection. Among the 19 patients with rectal tumors, 13 had preoperative radiotherapy: 4 long course (25 × 2 Gy) and 9 short course (5 × 5 Gy) treatments. Twenty-three patients (52%) received adjuvant chemotherapy after primary tumor resection.

### Liver and colorectal surgeries

Twenty-seven patients (61%) underwent major liver resection, involving three or more liver segments **(**Table 2**)**.

**Table 2:** Perioperative characteristics and postoperative complications for liver and colorectal resection

Liver Surgery			*N* = 44	
	Type of surgery	Right hepatectomy	10	23%
		Left hepatectomy	6	14%
		Extended right hepatectomy	8	18%
		Extended left hepatectomy	3	7%
		Sectionectomy	7	16%
		Bisegmentectomy	1	2%
		Segmentectomy	5	11%
		Wedge	4	9%
	30-day complications	Minor (Clavien < 3)	9	20%
		Major (Clavien ≥3)	14	32%
	30 and 90-day mortality		0	
	CCI (median, range)		29.3	8.7–60.8
	Liver specific complications	Liver failure	6	14%
		Biliary leak	9	20%
**Colorectal surgery**			*N* = 41	
	Type of surgery	Right colectomy	6	15%
		Left colectomy	3	7%
		Sigmoidectomy	12	29%
		Hartmann’s procedure	1	2%
		Rectal resection	4	10%
		LAR	15	37%
	30-day complications	Minor (Clavien < 3)	10	24%
		Major (Clavien ≥3)	6	15%
	30 and 90-day mortality		0	
	CCI (median, range)		20.9	8.7–83.4

*CCI* comprehensive complication index; *LAR* Low anterior resection.

Colorectal surgeries were performed in our institution (*n* = 33, 80%) or outwards (*n* = 8, 20%) **(**Table 2**)**. Colorectal resections were mainly performed by laparoscopy, with 28 laparoscopic approaches (68%), 9 open surgeries (22%), and one conversion (2%) due to adherent status with hepatic laceration during mobilization of the right colon. Data were missing for 3 patients with outwards operations.

No mortality occurred within 90 days after liver and colorectal surgery.

### Radiological and pathological response

Radiological response to chemotherapy according to the RECIST criteria was mainly partial response (*n* = 27, 61%) or stable disease (*n* = 12, 27%) **(**Table 3**).** One young patient with progressive disease at first evaluation received additional preoperative cycles of chemotherapy, which stopped further tumor growth and adjuvant treatment after hepatectomy. Liver histological response to neoadjuvant treatment was major or partial (TRG 1–3) in 23 patients (52%). Analysis of the non-tumoral liver parenchyma showed chemotherapy-related complications in 36 patients (81%) **(**Table 3**)**. The R0 resection rate was 61%.

**Table 3:** Radiological and pathological response to neoadjuvant chemotherapy and description of chemotherapy-related liver complications

		N = 44	
Radiological response (RECIST)	CR	0	0%
	PR	27	61%
	SD	12	27%
	PD	1	2%
	n.a.	4	9%
Pathological response ^a^	TRG 1	1	2%
	TRG 2	12	27%
	TRG 3	10	23%
	TRG 4	17	39%
	TRG 5	4	9%
Chemotherapy-related liver injury	Steatosis	26	59%
	Minimal	3	
	Grade 1	17	
	Grade 2	2	
	Grade 3	4	
	SOS	17	39%
	Grade 1	11	
	Grade 2	6	
	Fibrosis	16	36%
	F1	14	
	F2	2	
	Steatohepatitis	15	11%

*CR* Complete response; *PR* partial response; *SD* Stable disease; *PD* Progressive disease; *n.a* N

ot assessed; *TRG* Tumor regression grade; *SOS* Sinusoidal obstruction syndrome.

^a^ Pathological response according to Rubbia-Brandt et al.^13^ with report of the worst TRG score in case of multiple metastases with discordant response between lesions

Primary tumor response assessment according to Mandard revealed 7% (3/41) of major responses (TRG 1–2), 15% (6/41) of TRG 3 and 66% of poor response (TRG 4–5). Tissue for TRG analysis was not available in 5 (12%) patients. TNM stage was as following: 1 ypT0, 1 ypT1, 5 ypT2, 23 ypT3, 7 ypT4. There were 10 ypN0, 17 ypN1, 10 ypN2. R0 resection was achieved in 35 patients (95%). Tissue for TNM analysis was not available in 4 patients (10%).

### Survival

**Fig. 1:**
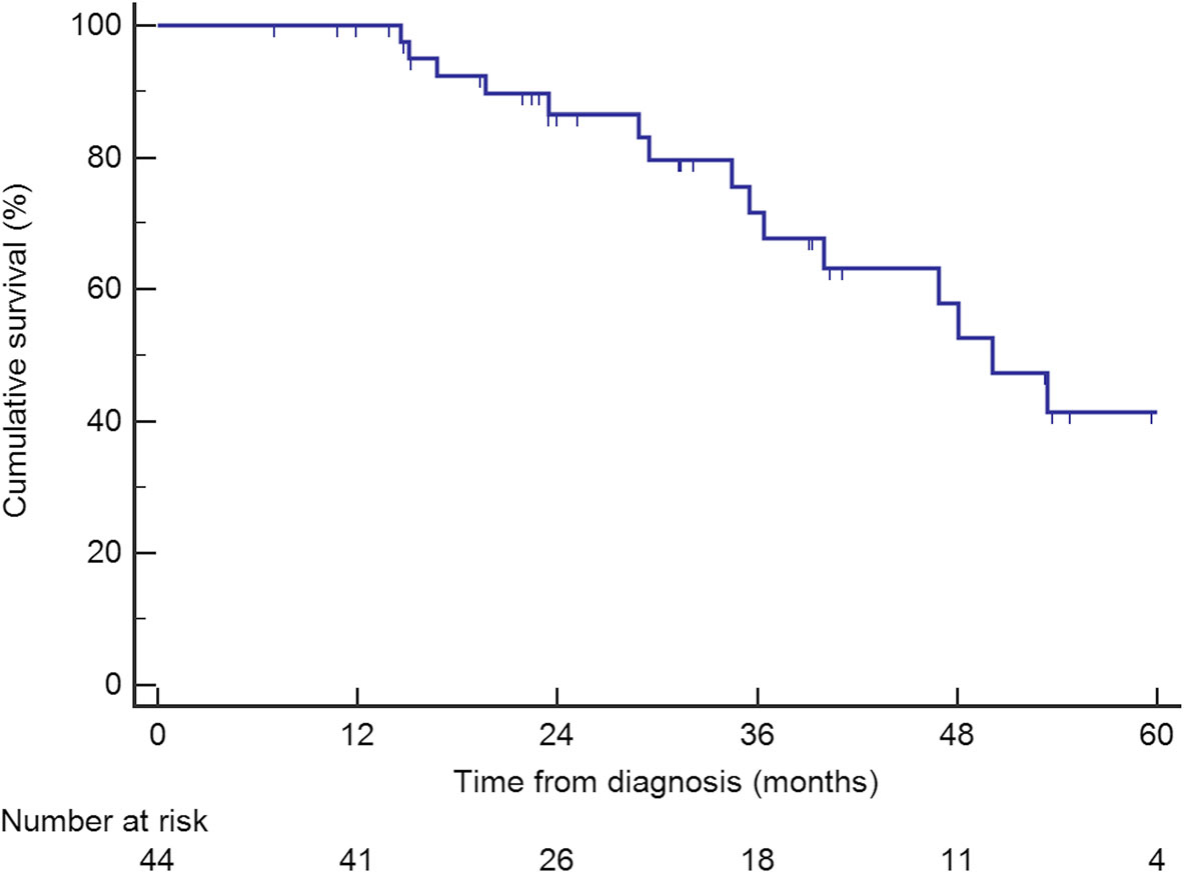
Overall survival from time of diagnosis of all patients who underwent liver resection for colorectal liver metastases as part of a reverse treatment

**Fig. 2:**
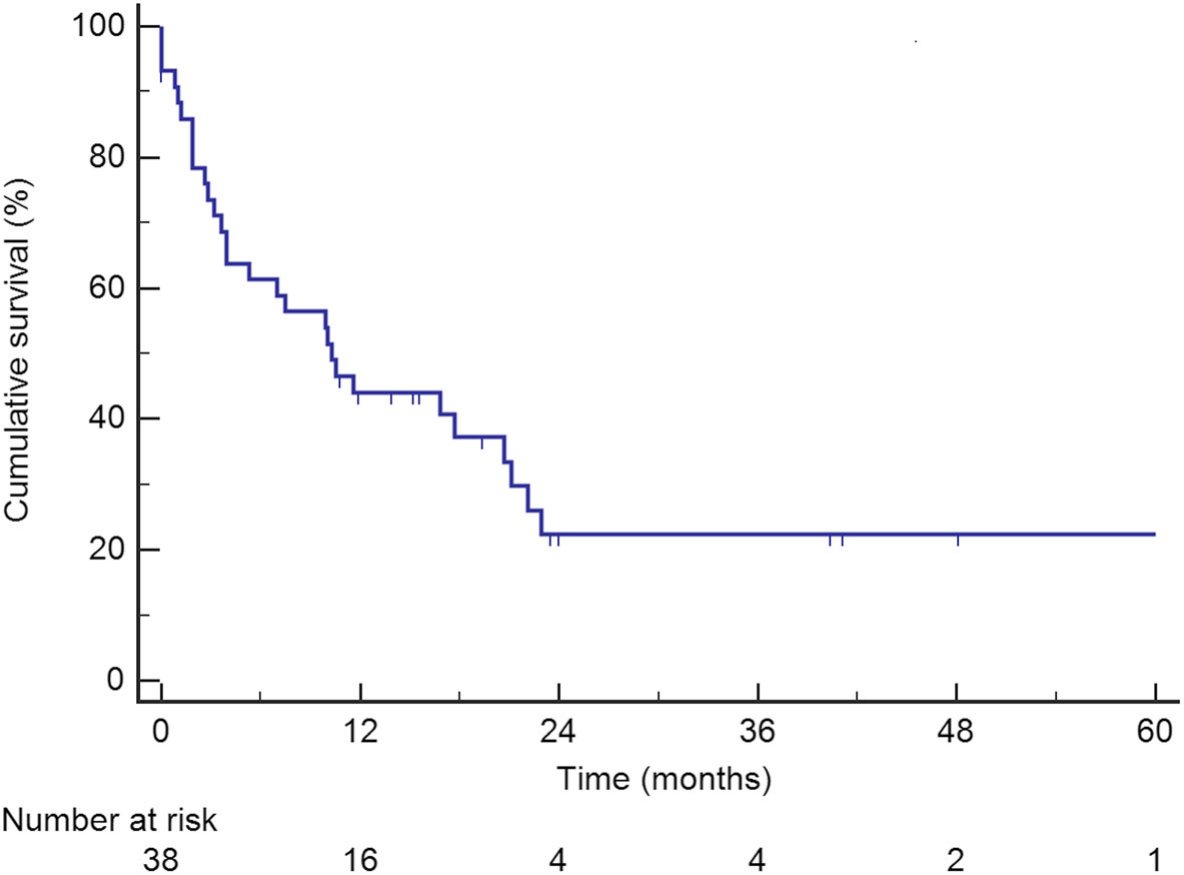
Disease-free survival from the time of primary tumor resection of patients who underwent liver resection for colorectal liver metastases as part of a reverse treatment

Median follow-up from time of diagnosis was 30.5 months. On an intention-to-treat basis, median OS from time of diagnosis was 50 months (95% CI 42–58), as shown in Fig. 1. Median DFS from time of primary tumor resection was 10 months (95% CI 5–15), as shown in Fig. 2.

## Discussion

This cohort study of patients undergoing liver-first approach for advanced synchronous CRLM revealed a high completion rate with 93% of patients who underwent the whole treatment sequence until primary tumor resection. The radiological tumor response rate showed 88% of partial response and stable disease according to RECIST criteria, and the pathological tumor response was 52% of major or partial response (TRG 1–3). A promising median overall survival of 50 months, with a 3-year overall survival of 59% was observed.

The completion rate of 93% in the present study was even higher than described in previous series (65 and 84%) [17–20]. Feared colonic complications such as occlusion or perforation during neoadjuvant treatment occurred only in 11% of the patients and did not preclude the completion of the whole sequence provided complications were adequately treated. The rates of colonic complications found in the literature ranged between 5 and 7% [17, 20]. Liver surgery was associated with a 52% morbidity, including 32% major complications. Previous studies reported morbidity ranging from 17 to 45% [18, 21–28], and major complication rates were 0 to 27.3% when reported [19, 22–26]. Our high complication rate could be explained by the aggressive surgical strategy for patients with high oncologic burden with 40% portal vein embolization and 61% major hepatectomies, compared to 11–60% portal vein embolization [18, 21, 22, 28, 29] and 36–89% major hepatecomies [18–20, 22–26, 29, 30] in other reports. However, there was no mortality within 90 postoperative days, while the highest reported mortality rate of 2% in the series from Welsh et al. [19]. Morbidity after colorectal resection was 39% in the present study, with 15% major complications, concordant with other series reporting 16.7 to 44.4% overall morbidity [23, 24, 26].

Radiological response of CRLM to neoadjuvant treatment was predominantly partial response (61%), followed by stable disease (27%). These results were similar to those found in the literature with 3 studies reporting 5–8% complete response, 68–83% partial response, 0–30% stable disease and 8% progressive disease [18, 26, 30]. Pathological response of CRLM to neoadjuvant treatment was major or partial (TRG 1–3) in more than half of the patients (52%). A previous study also reported the histological response rate among patients undergoing reverse approach, with 93% (27/29) of TRG 1–3 [31]. The lower histological response observed in the present study is difficult to compare as various regimens of neodajuvant chemotherapies were used because of referred cases. Histological response of the primary tumors were major (TRG 1–2) in 7% and poor (TRG 4–5) in 66%, compared to 35% in each group in the study of Gervaz et al. with standardized chemotherapy [31]. R0 resection was achieved in 61% of the patients, which is comparable with other studies reporting compete resection rates of 50–80% [18, 26, 27, 32].

The OS rates were 59 and 39% at 3 and 5 years, respectively, with a median OS of 50 months. These results were in line with other reports, with a 3 and 5-year OS of 30 to 89% and 30 to 72%, respectively [19, 21–24, 26, 27, 30, 33]. Nine studies compared the outcomes of reverse and classical treatments with similar results in both groups regarding OS. However these findings need to be balanced with the fact that there were significantly more liver metastases [19, 22, 26], larger lesions [27, 28] and more bilobar spread in the liver-first group [25]. Nevertheless, two studies showed similar OS in both groups after propensity score matching [27, 29]. The DFS at 3 years was 25% in the present study, which is comparable with DFS rates reported in the literature, ranging from 0 to 31% [18, 26, 27, 33].

Among limitations, the retrospective analysis of prospectively collected data in a single institution presents its inherent risks of bias of patient selection, missing data, and loss to follow up. Patients included in the reverse treatment were highly selected and only patients who underwent liver resection for synchronous CRLM before removal of the primary tumor were included. Moreover, as a tertiary referral center, some patients were addressed from other centers after favorable response to chemotherapy. Accordingly, data on patients with intention to reverse approach but who failed to undergo liver resection were missing. In the same way, the exact number of patients who were initially considered as unresectable and who presented a significant response allowing a liver resection was not available. Despite patients’ heterogeneity in terms of chemotherapy regimens or tumor burden, no subgroup analysis was performed due to the limited number of patients. The main weakness of such a study is the absence of control group. However, as 75% of liver metastasis were unresctable initially, the only real control group should not be patient with “classical” treatment sequence (colon first), but patients with palliative chemotherapy only. Similar observation may be done on other series [19, 22, 26–28], with some cases of smaller liver metastasis allowing to choose between reverse or classical treatment, which was not the case in the present series.

## Conclusions

In conclusion, the reverse treatment is safe and feasible with a high rate of patients undergoing the whole process. With high rate of tumor response during chemotherapy, the reverse approach allows resection of both liver and pulmonary metastases, as well as resection of the primary tumor, with a promising long-term survival for highly selected patients with advanced synchronous colorectal liver metastases. Therefore, patients with synchronous colorectal liver metastases, even with initially unresectable disease, should be discussed in multidisciplinary board to assess the feasibility of such a reverse treatment.

## Data Availability

The datasets generated and/or analysed during the current study are not publicly available due to the terms agreed with the local ethics committee with full access restricted to CDP and DR. They are available from the corresponding author on reasonable request.

## Abbreviations

CCI: Comprehensive complication index
CEA: Carcino embryogenic antigen
CRLM: Colorectal liver metastases
DFS: Disease-free survival
FRL: Future remnant liver
LAR: Low anterior resection
OS: Overall survival
RECIST: Response evaluation criteria in solid tumor
SOS: Sinusoidal obstruction syndrome
TRG: Tumor regression grade
